# Volatile diterpene emission by two Mediterranean *Cistaceae* shrubs

**DOI:** 10.1038/s41598-018-25056-w

**Published:** 2018-05-01

**Authors:** A. M. Yáñez-Serrano, L. Fasbender, J. Kreuzwieser, D. Dubbert, S. Haberstroh, R. Lobo-do-Vale, M. C. Caldeira, C. Werner

**Affiliations:** 1 Ecosystem Physiology, University Freiburg, Freiburg, Germany; 2 Centro de Estudos Florestais, Instituto Superior de Agronomia, Universidade de Lisboa, Tapada da Ajuda, Lisboa, Portugal

## Abstract

Mediterranean vegetation emits a wide range of biogenic volatile organic compounds (BVOCs) among which isoprenoids present quantitatively the most important compound class. Here, we investigated the isoprenoid emission from two Mediterranean Cistaceae shrubs, *Halimium halimifolium* and *Cistus ladanifer*, under controlled and natural conditions, respectively. For the first time, diurnal emission patterns of the diterpene kaurene were detected in real-time by Proton-Transfer-Reaction-Time-of-Flight-Mass-Spectrometer. Kaurene emissions were strongly variable among *H*. *halimifolium* plants, ranging from 0.01 ± 0.003 to 0.06 ± 0.01 nmol m^−2^ s^−1^ in low and high emitting individuals, respectively. They were in the same order of magnitude as monoterpene (0.01 ± 0.01 to 0.11 ± 0.04 nmol m^−2^ s^−1^) and sesquiterpene (0.01 ± 0.01 to 0.52 nmol m^−2^ s^−1^) emission rates. Comparable range and variability was found for *C*. *ladanifer* under natural conditions. Labelling with ^13^C-pyruvate suggested that emitted kaurene was not derived from *de novo* biosynthesis. The high kaurene content in leaves, the weak relationship with ecophysiological parameters and the tendency of higher emissions with increasing temperatures in the field indicate an emission from storage pools. This study highlights significant emissions of kaurene from two Mediterranean shrub species, indicating that the release of diterpenes into the atmosphere should probably deserve more attention in the future.

## Introduction

Vegetation is the largest global emission source of biogenic volatile organic compounds (BVOCs) with an estimated 1000 Tg of carbon yr^−1^^1^, having a profound impact on the atmosphere’s chemistry and biogenic secondary aerosol formation. Quantitatively, the most important group of BVOCs are isoprenoids, which fulfil many essential functions in plants. At the (sub-) cellular level, they can protect against oxidative damage^2–4^ while at plant and ecosystem level, isoprenoids can serve as signalling compounds within plant-plant and plant-insect interactions as well as protection against biotic and abiotic stress^5,6^. Isoprenoids can be synthesized either via the plastidic non-mevalonate (methylerythrol-phosphate, MEP) pathway or the cytosolic mevalonate (MVA) pathway^7^. Emission of volatile isoprenoids can result from *de novo* biosynthesis, which is directly linked to photosynthetic CO_2_ assimilation, or from the release from specialized structures serving as storage pools of isoprenoids. Henceforth, when isoprenoid emissions are controlled by *de novo* biosynthesis, the driving abiotic factors are solar radiation as a factor controlling net CO_2_ assimilation, and temperature, since it influences (i) enzymatic processes and consequently *de novo* isoprenoid biosynthesis, and (ii) the volatility of stored isoprenoids. Importantly, *de novo* biosynthesis ceases when temperatures are higher than the optimum temperature of enzymes involved in isoprenoid biosynthesis, whereas emissions from storage pools may further increase^8,9^. In addition, biotic stresses such as herbivory or pathogen attack^10^, and other abiotic factors such as ambient ozone mixing ratios^11^, water stress, or soil fertility may indirectly influence isoprenoid emissions^12,13^ by affecting plant physiology and thereby the enzymatic production of isoprenoids.

In addition to highly volatile isoprenoids, such as isoprene (C_5_), monoterpenes (C_10_), and sesquiterpenes (C_15_), plants also synthesize larger isoprenoids such as diterpenes (C_20_), sesterterpenes (C_25_), triterpenes (C_30_) and even more complex compounds, which are overall assumed to be non-volatile^14–17^. High molecular weight isoprenoids carry a plethora of other essential functions as they are involved in crucial processes such as photosynthesis (e.g. carotenoids, chlorophylls), membrane integrity (e.g. tocopherol), as well as plant growth and development (e.g. the phytohormones gibberellins, cytokinins)^15,18–21^. In particular, diterpenes (C_20_ isoprenoids) have been reported to play an important role in ecological interactions between plants and other organisms, such as signalling and defence against herbivores^6,22–25^; in addition, they provide an essential building block for chlorophyll, which is a diterpene conjugate, thus, diterpenes are also involved in light-dependent reactions of photosynthesis. Moreover, diterpenes can serve as growth regulators controlling crucial processes, namely germination, cell elongation and division, or flower and fruit development^15,18,20,24^. So far diterpenes were generally considered as non-volatile compounds, even though first records of emissions in three plant species were reported^19,26,27^. However, these measurements were sampled with low time resolution based on pre-concentration techniques, and thus very little is known on the dynamics of diterpene emissions or their role in the atmosphere.

To shed new light on the potential role of the emission of isoprenoids with high molecular weight masses, we selected two Mediterranean shrubs from the Cistaceae family. These plant species possess a very active secondary metabolism generating numerous metabolites, including isoprenoids of high molecular weight^15^. It is assumed that these compounds are involved in physiological and ecological processes, that most likely contribute to plant survival in such harsh environments^28^. Therefore, we hypothesize that these characteristic Mediterranean shrubs *Cistus ladanifer* and *Halimium halimifolium* emit a wide variety of isoprenoids. In particular, considering that these species contain high molecular weight isoprenoids such as diterpenes, we hypothesize that they may also emit such large isoprenoids into the atmosphere. To assess these hypotheses, we investigated the dynamics of isoprenoid emissions of *C*. *ladanifer* and *H*. *halimifolium* under natural and controlled conditions, respectively. A particular emphasis was placed on the emission patterns of high molecular weight compounds, which we discuss in terms of their emission sources, function, and possible ecological relevance.

## Results

### Diurnal cycles of isoprenoid emissions by *H*. *halimifolium* under controlled conditions

Substantial emission of monoterpenes, sesquiterpenes and even the diterpene kaurene were found in *H*. *halimifolium*. Despite controlled environmental conditions, isoprenoid emission rates greatly varied among the seven individuals with a group of plants showing high emission rates and another group with low emission rates. Since the emission rates of the two groups significantly differed for all compound classes (p < 0.001; with the exception of sesquiterpenes, where only one out of seven plants was above the mean emission rate), we classified the plants into the groups of high and low emitters. Plants exceeding the mean emission rates for a specific group of isoprenoids were classified as high emitters, plants showing lower than mean emission rates were defined as low emitters. Mean emission rates were 0.008 ± 0.005 nmol m^−2^ s^−1^, 0.032 ± 0.028 nmol m^−2^ s^−1^, 0.062 ± 0.104 nmol m^−2^ s^−1^ and 0.022 ± 0.016 nmol m^−2^ s^−1^ for isoprene, monoterpenes, sesquiterpenes and kaurene, respectively.

**Figure 1:**
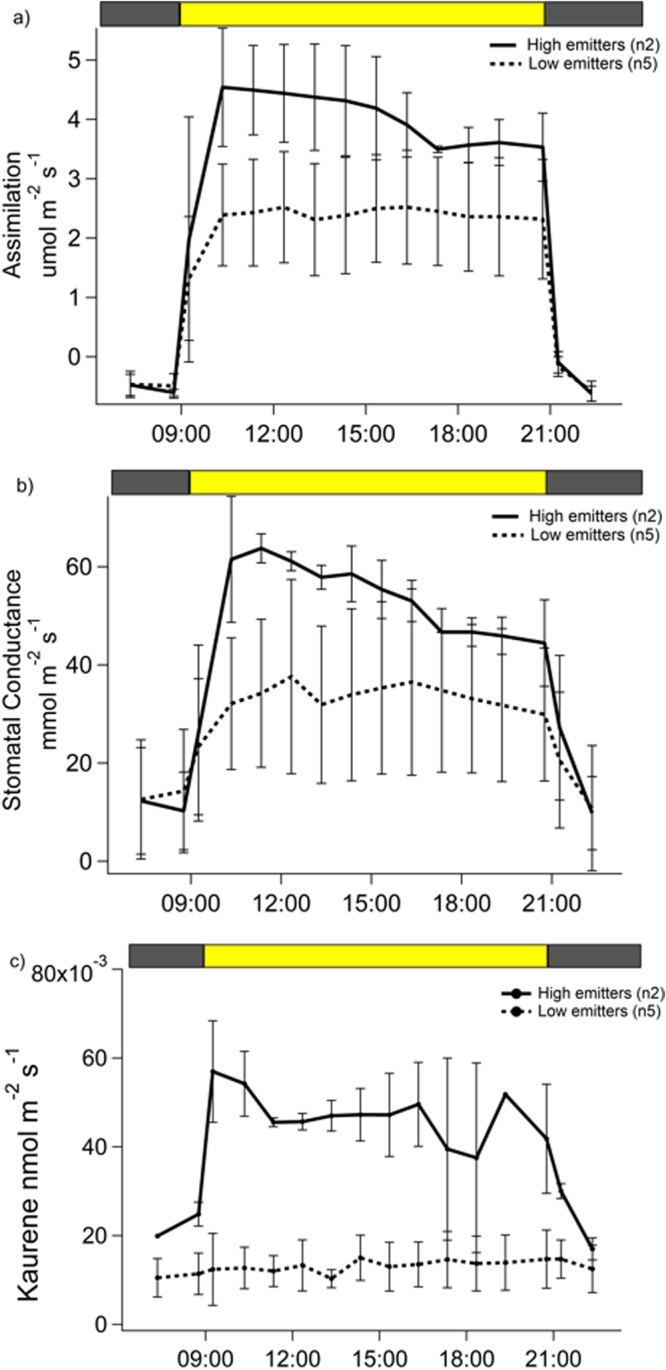
Diurnal cycles of kaurene emission rates (**a**) CO_2_ assimilation rate (**b**) and stomatal conductance (**c**) by seven individuals of *H*. *halimifolium*. The thick line represents the high emitter group (n = 2) and the dashed line represents the low emitter group (n = 5). Error bars indicate one standard deviation. The top bar indicates the light (yellow) and dark (grey) periods.

Plants with high isoprenoid emission rates were physiologically more active, with higher net CO_2_ assimilation rate and stomatal conductance (4.0 ± 0.4 µmol m^−2^ s^−1^ and 54.1 ± 7.1 mmol m^−2^ s^−1^, respectively) during the light period than low emitters (2.4 ± 0.1 µmol m^−2^ s^−1^ and 33.7 ± 2.3 mmol m^−2^ s^−1^, respectively) (Fig. 1a,b). Interestingly, when considering the emission rates of kaurene (Fig. 1c), the pattern of the diel cycles differed between the high and low emitters. Whereas the high emitters showed a sharp increase in kaurene emissions from 0.02 ± 0.01 nmol m^−2^ s^−1^ in the dark, to 0.06 ± 0.01 nmol m^−2^ s^−1^ during the light period, no such increase was observed for the low emitters; their kaurene emission rates remained constant at 0.01 ± 0.01 nmol m^−2^ s^−1^. Notably, night-time emission of kaurene was clearly detectable and comparable in both high and low emitting plants.

**Figure 2:**
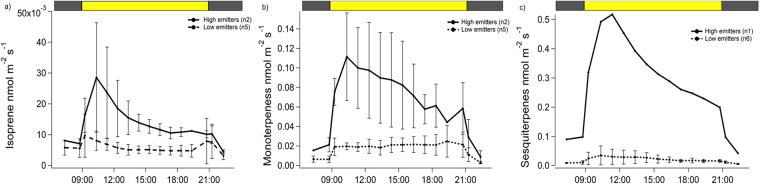
Diurnal cycles of the emission rates of isoprene (**a**), monoterpenes (**b**) and sesquiterpenes (**c**) by seven individuals of *H*. *halimifolium*. The thick line represents the high emitter group and the dashed line the low emitter group. Error bars indicate one standard deviation. Note that for sesquiterpenes only one plant represents the high emitter group. The top bar indicates the light (yellow) and dark (grey) periods.

For the other isoprenoids released by *H*. *halimifolium*, generally highest emission rates occurred before noon (Fig. 2). Particularly, monoterpene and sesquiterpene emission rates of the high emitter group increased sharply at the beginning of the light period, reaching a maximum at 10:00 h for monoterpenes and at 11:00 h for sesquiterpenes. Thereafter, monoterpene and sesquiterpene emission rates steadily decreased over the day reaching early morning values, comparable to the emission rates of the low emitters. Nevertheless, low emission rates persisted in both groups during the dark period, suggesting a possible release of these compounds from storage pools during night. Pronounced differences in emission rates were observed for different isoprenoids (Fig. 2): isoprene emission ranged from 0.003 ± 0.001 nmol m^−2^ s^−1^ (low emitters) to 0.023 ± 0.02 nmol m^−2^ s^−1^ (high emitters), whereas emission rates of monoterpenes and sesquiterpenes of the strongly emitting plants were significantly higher (0.11 ± 0.04 nmol m^−2^ s^−1^ and 0.52 nmol m^−2^ s^−1^, respectively). Although all plants were kept under similar environmental conditions and no visible pest or damaged was observed, biotic factors could have caused such high sesquiterpene emissions.

**Figure 3:**
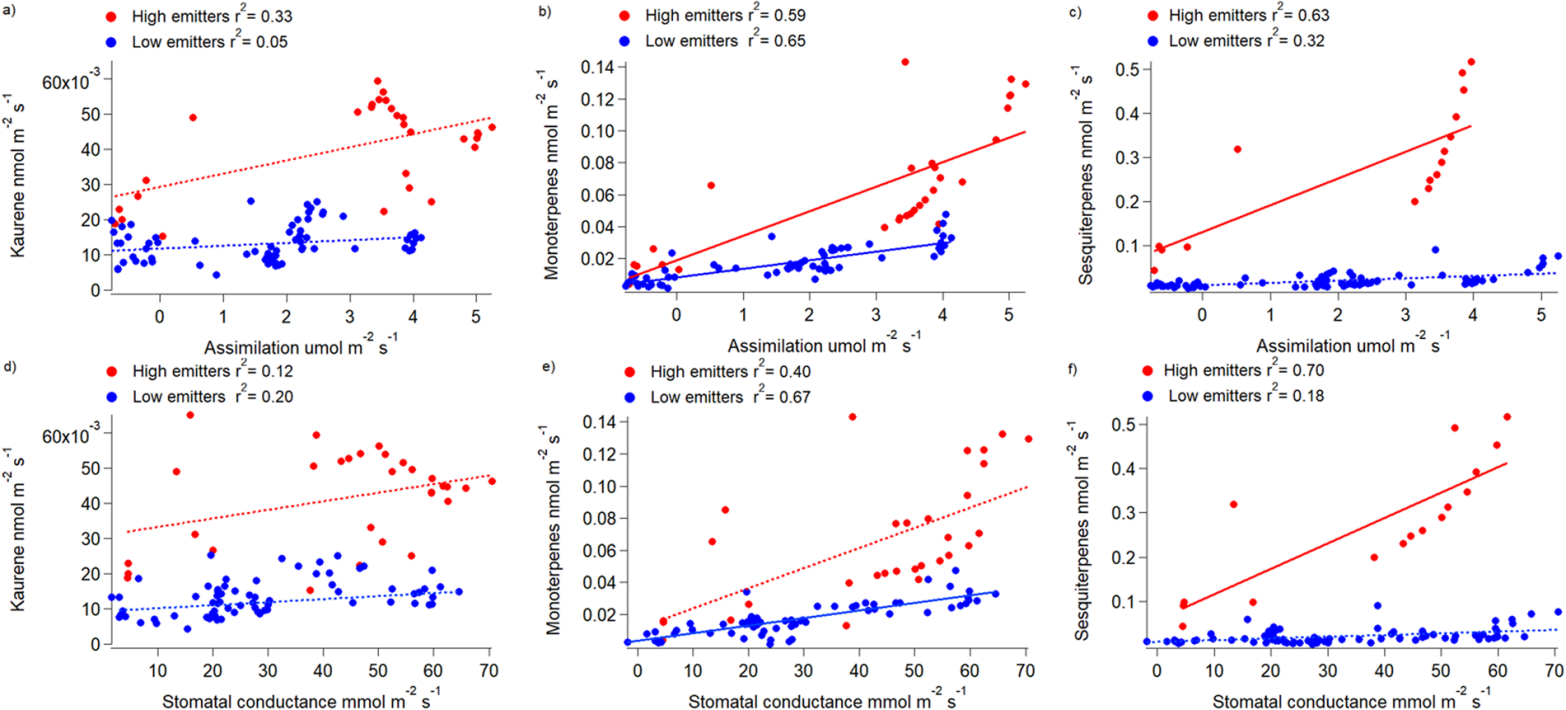
Linear correlations of CO_2_ assimilation rate (upper part) and stomatal conductance (bottom part) with kaurene (**a**,**d**), monoterpenes (**b**,**e**) and sesquiterpenes (**c**,**f**) emission rates for the diurnal cycles measurements of seven individuals of *H*. *halimifolium*. Red dots represent the high emitter group and blue dots the low emitter group. Thick lines represent a correlation and dashed lines represent a lack of correlation. Note that for sesquiterpenes only one plant represents the high emitter group. R^2^ are shown in the legend, all correlations were statistically significant (p < 0.001) except for the relationship between kaurene emission rates and CO_2_ assimilation rates.

To identify ecophysiological parameters that could control isoprenoid emission rates correlation analyses were performed (Fig. 3). Rates of kaurene emissions did not significantly correlate with CO_2_ assimilation rates or stomatal conductance (p > 0.001, Fig. 3a,d). In contrast, monoterpene emissions significantly correlated with both, assimilation rates and stomatal conductance (p < 0.001). Net CO_2_ assimilation explained 59% and 65% of the variability in monoterpene emissions, whereas stomatal conductance explained 40% and 67% of the variability, for high and low emitters, respectively (Fig. 3b,e). Sesquiterpene emission of the only high emitter showed high correlation with CO_2_ assimilation (r^2^ = 0.63) and stomatal conductance (r^2^ = 0.70), while correlations for the low emitters were weak (Fig. 3c,f). The different relationships between isoprenoid emission rates and the ecophysiological parameters of high and low emitters, might indicate that monoterpenes and sesquiterpenes were emitted from both, *de novo* biosynthesis plus release from storage pools.

### Isoprenoid content in leaves of *H*. *halimifolium*

**Figure 4:**
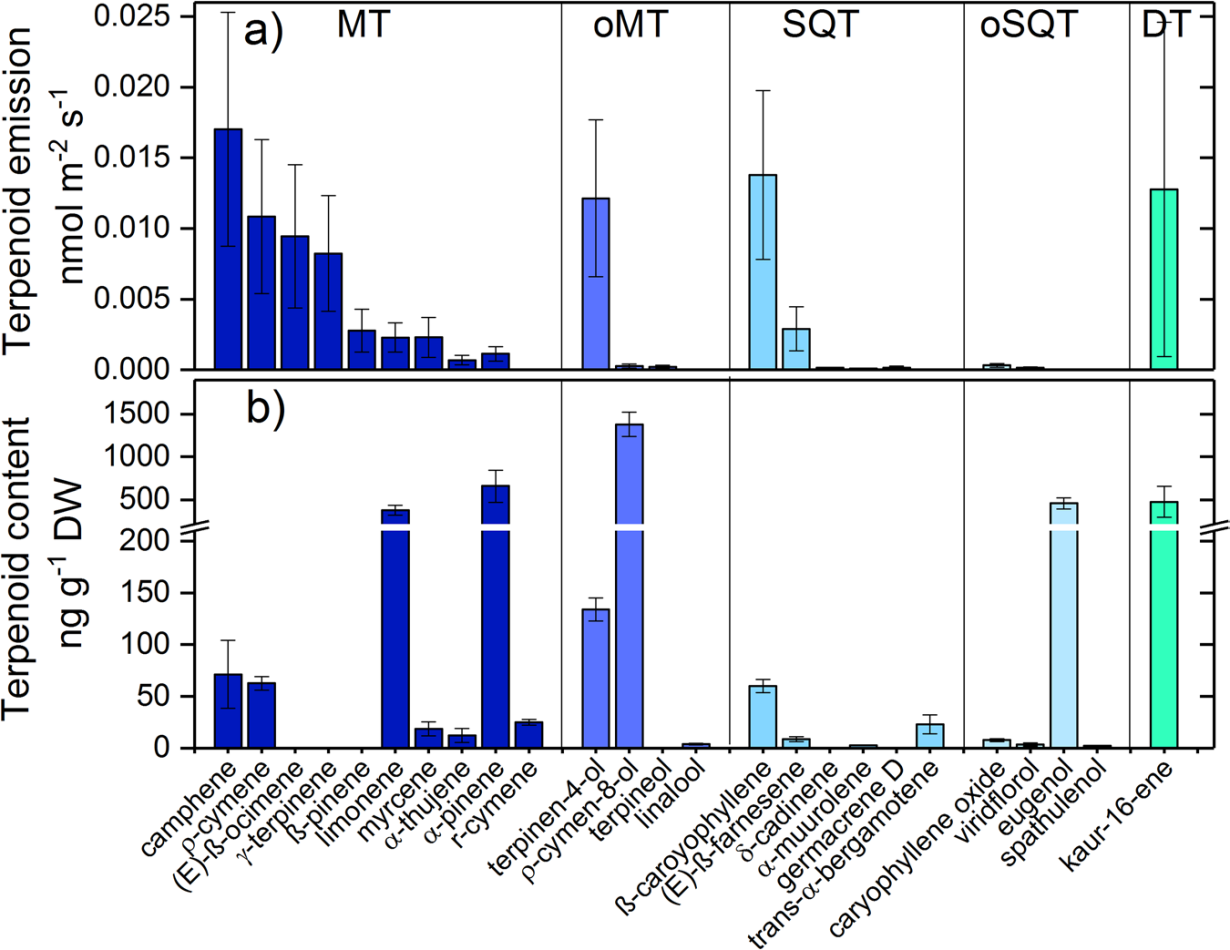
Isoprenoid emission rates (**a**) and leaf isoprenoid concentrations (**b**) of *H*. *halimifolium* determined with TD-GC-MS. Leaf isoprenoid concentration was determined from five *H*. *halimifolium* individuals. For the isoprenoid emission measurements three different *H*. *halimifolium* individuals were used. MT stands for monoterpenes, oMT stands for oxygenated monoterpenes, SQT stands for sesquiterpenes, oSQT stands for oxygenated sesquiterpenes and DT stands for diterpenes.

To further characterize individual isoprenoids released by *H*. *halimifolium* TD-GC-MS analysis was performed. The quantitatively most important isoprenoids emitted from this plant species were camphene, β-caryophyllene, kaurene, terpinen-4-ol, ρ-cymene, (E)-β-ocimene and γ-terpinene (Fig. 4a). Interestingly, the concentrations of isoprenoids in leaves partially differed from the emission pattern, which could indicate the source of emission. For instance, the high abundance of camphene and ρ-cymene in the leaves (Fig. 4b) was in line with the observed emission rates of these compounds, indicating that their emission was most probably driven by release from storage pools. However, (E)-β-ocimene and γ-terpinene were not abundant in significant amounts in leaf tissue, suggesting that their strong emission might be driven by *de novo* biosynthesis. In leaves, ρ-cymen-8-ol, limonene, and α-pinene were by far the most abundant monoterpenoids, although their emission was relatively low. The most abundant sesquiterpenes in the leaves, β-caryophyllene and (E)-β-farnesene, were well correlated with their high emission rates. Interestingly, the very high abundance of eugenol did not drive any emission of this oxygenated sesquiterpenoid. Importantly, kaurene was found in high amounts in the leaves of *H*. *halimifolium* in accordance with the high emission rates of this diterpene, and suggesting that emissions were derived from storage pools.

### ^13^C-Labelling experiments

All ^13^C-labelling experiments which resulted in a detectable isotopic signal in isoprenoids are shown in Table 1. All plants in this experiment reached leaf CO_2_ assimilation rates of 2–3 µmol m^−2^ s^−1^ and stomatal conductance of 20–40 mmol m^−2^ s^−1^ during the light period. The theoretical natural abundance of the first isotopologue (*i*.*e*. abundance of the molecule containing one ^13^C-atom relative to the light molecule exclusively containing ^12^C) is: 5.2% for isoprene, 9.7% for monoterpenes, 13.8% for sesquiterpenes and 17.5% for diterpenes (*sensu* Isotope library, PTR-MS Viewer, Ionicon Analytic, Austria). Our measured natural abundance of the isoprenoid isotopologues matched well the theoretical expected percentage, supporting the accuracy and precision of our measurements. Both, monoterpenes and sesquiterpenes quickly incorporated ^13^C after 30 minutes of labelling with ^13^C-pyruvate fed to the branches, amounting to 21.2 and 24.6% for monoterpenes and sesquiterpenes, respectively, even though labelling of sesquiterpenes showed a high variability (Table 1). In contrast, kaurene did not show any ^13^C incorporation as the abundance of the ^13^C- isotopologue remained constant during the labelling experiment. Thus, in agreement with the data obtained for isoprenoid leaf content and emissions (Fig. 4), the labelling experiments supported the assumption of emissions from storage pools plus *de novo* biosynthesis for mono- and sesquiterpenes, while emission of kaurene seemed to originate solely from storage pools.

**Table 1:** Change in the proportion (%) of the heavy isotopologue (^13^C) molecules with respect to the parent monoterpenes, sesquiterpenes and diterpenes for the labelling experiments.

Compound	Theoretical 1^st^ isotopologue natural abundance (%)	Measured 1^st^ isotopologue natural abundance (%)	1^st^ isotopologue abundance 30 mins after labelling (%)	Sample number
Monoterpenes	9.7	9.1 ± 0.6	21.2 ± 8.3	8
Sesquiterpenes	13.8	13.3 ± 1.5	24.6 ± 10.5	5
Diterpenes	17.5	16.3 ± 0.1	16.8 ± 0.7	6

Ten different (only detectable isotopic signals for each isoprenoid are show *H*. *halimifolium* individuals were used for this experiment).

### Kaurene emissions by *C*. *ladanifer* under natural conditions

**Figure 5:**
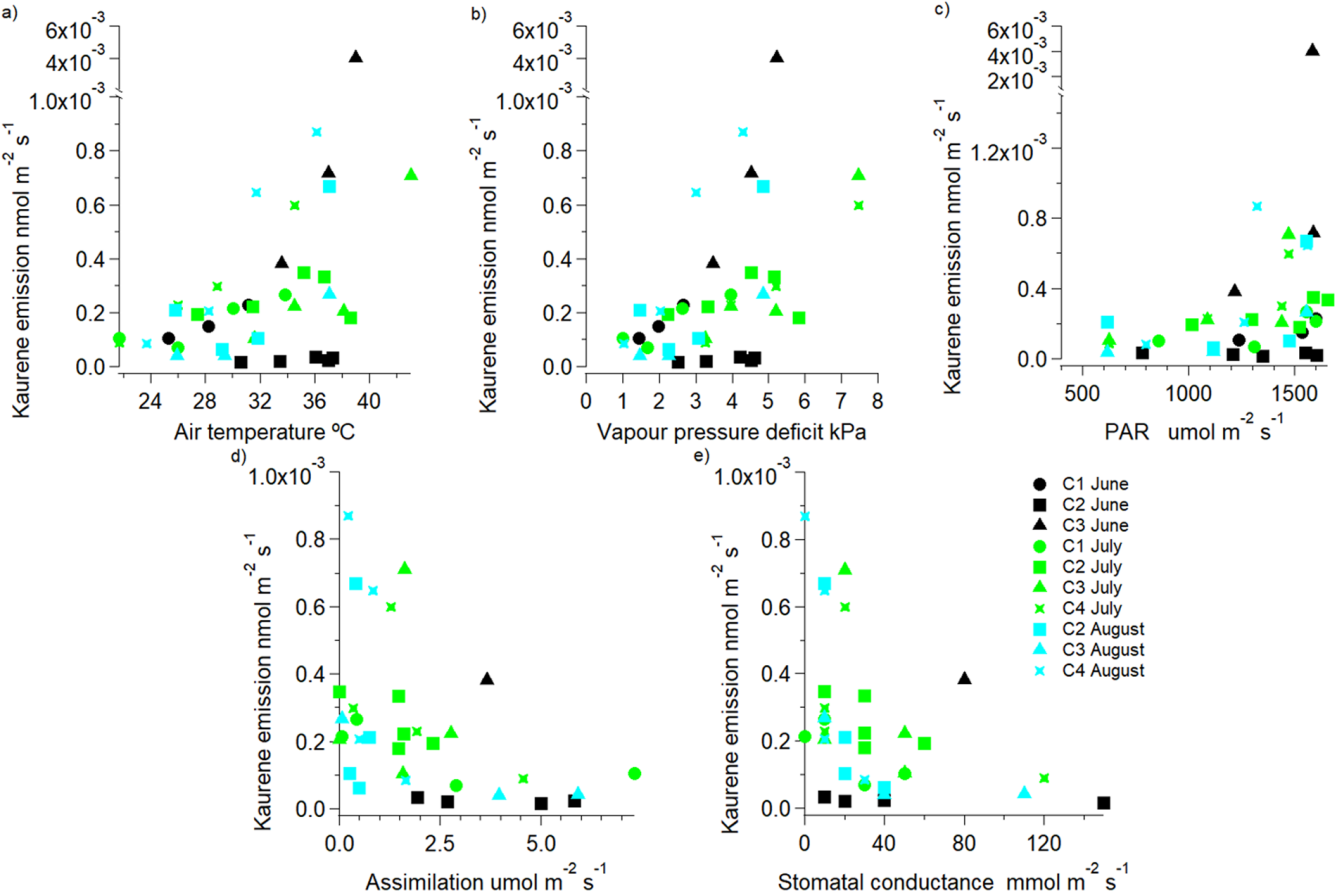
Kaurene emission rates by *C*. *ladanifer* under natural conditions plotted against air temperature (**a**) vapour pressure deficit (**b**) photosynthetically active radiation (PAR, (**c**) CO_2_ assimilation (**d**) and stomatal conductance (**e**) for four *C*. *ladanifer* plants (C1–C4) analysed in the field during June (black), July (green) and August (blue) 2017.

Measurements of kaurene emission from leaves of *C*. *ladanifer* were performed in the field to assess its relevance and magnitude under natural conditions. Our field measurements clearly indicated that *C*. *ladanifer* shrubs emit kaurene under natural conditions (Fig. 5). Similar to the emission patterns of *H*. *halimifolium* under controlled conditions, kaurene emission from *C*. *ladanifer* leaves exhibited a high variability. Emission rates ranged from 0.0001 to 0.006 nmol m^−2^ s^−1^, which is somewhat lower than the emissions rates observed for *H*. *halimifolium* (0.007 nmol m^−2^ s^−1^ to 0.067 nmol m^−2^ s^−1^). Nevertheless, it is important to note that these emission rates might be underestimated, since kaurene losses due to ozonolysis, *i*.*e*., reactions with OH-radicals, as well as adsorption of kaurene onto tubing surfaces might have occurred in the field.

A tendency of higher kaurene emissions along with the diurnal increase in air temperature, vapour pressure deficit (VPD) and to a lower extent photosynthetically active radiation became evident for each measured shrubs (Fig. 5). Clear differences in emission rates at the measurement dates were not visible, which may be due to comparable high temperatures on each sampling day, from June to August (maximum daily temperature recorded were 36.7 ± 2.7 °C for June, 38.7 ± 4.2 °C for July, and 35.5 ± 1.9 °C for August). Interestingly, kaurene emission rates were higher at lower stomatal conductance and net CO_2_ assimilation rates, which declined with the diurnal rise in temperature (note that not for all plants screened for kaurene emission plant physiological data are available).

## Discussion

Here, we demonstrate that two characteristic Mediterranean shrub species of the Cistaceae family, *H*. *halimifolium* and *C*. *ladanifer*, emit the diterpene kaurene into the atmosphere. The observed emission patterns of kaurene were comparable to those of monoterpenes and sesquiterpenes. To the best of our knowledge, only three studies have reported kaurene emissions from vegetation so far, *i*.*e*. from *Cryptomeria japonica* and *Chamaecyparis obstusa*, the dominant conifer trees in Japan^19,26^, and from the moss *Physcomitrella patens*^27^. Therefore, BVOC studies of different species or ecosystems have rarely addressed diterpene emissions eventually because of former technical difficulties to detect diterpenes due to the low volatility and high molecular weight^16,17^. On the other hand, the Mediterranean plant species studied so far may either not emit this diterpene, or the compound was just overlooked. We speculate that diterpene emission may be restricted to plant species with a highly active secondary metabolism as shown for *Cryptomeria japonica*^29,30^, *C*. *ladanifer*^15,31^, and *H*. *halimifolium*^32,33^.

The observed kaurene emission rates of *H*. *halimifolium* were on average 0.034 nmol m^−2^ s^−1^ (equal to 0.53 µg g_dw_^−1^ h^−1^) matching those reported earlier for *C*. *japonica and C*. *obstusa*^26^. Furthermore, monoterpene emission rates of *H*. *halimifolium* were comparable with those of other Mediterranean shrubs, such as *Cistus albidus* or *Cistus monspeliensis* (0.3–1.03 µg g_dw_^−1^ h^−1^)^31^ and matched those reported earlier for *H*. *halimifolium*^34^, whereas the sesquiterpene emission rates were slightly higher than those found for other Cistaceae species, but still in the same order of magnitude (0.6–0.63 µg g_dw_^−1^ h^−1^)^31^. In agreement with earlier studies on species of the Cistaceae^31^ family and similar to other Mediterranean species, which are rather characterised by high monoterpene and sesquiterpene emissions^35^, isoprene emission rates were very low (ca. 0.013 nmol m^−2^ s^−1^ or 0.05 µg g_dw_^−1^ h^−1^), thus, classifying *H*. *halimifolium* as a low isoprene emitter.

Isoprenoid emission might be driven by a temperature dependent release of leaf internally stored compounds and/or from *de novo* biosynthesis, *i*.*e*. direct emissions after production without long-term storage in particular structures. Our results indicate that kaurene emissions in *H*. *halimifolium* and *C*. *ladanifer* are not driven by *de novo* biosynthesis but mainly by the release from storage pools, which contrasts the emission pattern of monoterpenes and sesquiterpenes. Therefore, we postulate that temperature is a major factor determining kaurene emissions, as this is the main driver for release from storage pools^36^.

The assumption that kaurene emissions and some other isoprenoids derived from storage pools is based on the high abundance of kaurene and other isoprenoids in leaves of *H*. *halimifolium* coinciding with observed emission pattern. These included monoterpenes and monoterpenoids (e.g. camphene, ρ-cymene, terpinen-4-ol) and sesquiterpenes (β-caryophyllene, (E)-β-farnesene). In contrast, some monoterpenes ((E)-β-ocimene, γ-terpinene) were less abundant in leaves but were strongly emitted, suggesting *de novo* biosynthesis and direct emission after production. Moreover, kaurene emission from leaves of *H*. *halimifolium* clearly occurred during night, which strongly suggests a release from storage pools. In addition, kaurene emissions were not directly linked to CO_2_ assimilation rates, differing from monoterpenes and sesquiterpenes that were better correlated with ecophysiological parameters.

To further disentangle the origin of the isoprenoids emitted by *H*. *halimifolium*, we performed ^13^C-labelling studies by applying position labelled ^13^C-pyruvate, which has been proven to rapidly be incorporated into freshly synthesized BVOCs^34^. The observed incorporation of ^13^C derived from pyruvate into the emitted monoterpenes and sesquiterpenes suggests that these compounds are at least partially derived from *de novo* biosynthesis. This is supported by the clear discrepancy between considerable emissions of (E)-β-ocimene, γ-terpinene and β-pinene, and the low abundance of these compounds in the leaves. It is generally thought that the biosynthesis of monoterpenes occurs via the MEP pathway within the plant chloroplasts, which directly depends on the plastidic availability of pyruvate and glyceraldehyde-3-phosphate. Therefore, it must be assumed that the pyruvate fed through the transpiration stream to the leaves, was taken up by mesophyll cells and subsequently channelled into chloroplasts^37^. On the other hand, sesquiterpenes are mainly synthesized through the cytosolic MVA pathway^21,38–41^ and the incorporated ^13^C of pyruvate most likely entered this pathway after conversion of pyruvate into acetyl-CoA^7^. In contrast to monoterpenes and sesquiterpenes, no sign of ^13^C-incorporation was obtained for kaurene. This strongly indicates that kaurene emission was mainly mediated by the release from storage pools as also suggested by the high kaurene abundance in leaves.

The important role of temperature for the release of isoprenoid from storage pools is further supported by the field measurements that showed a clear tendency of higher kaurene emission rates at higher temperatures for *C*. *ladanifer* under natural conditions. This tendency further indicates the important role of temperature for isoprenoid emissions from storage pools, in accordance with earlier work^16^ on temperature effects for kaurene emission rates. Furthermore, in the field, kaurene emission rates were highest at high temperatures when rates of net CO_2_ assimilation and stomatal conductance were low. During the Mediterranean summer, high CO_2_ assimilation often occurs only in the early morning hours. Thereafter, temperature and VPD increases inducing stomatal closure to protect plants against excessive water loss on very hot and dry summer days. Therefore, this is another hint of evidence that kaurene emissions were mainly driven by the diurnal increase in temperature and to a lesser extent by plant physiological processes and delivers further support to the premise that diterpene emissions may be mainly derived from storage pools and not by *de novo* biosynthesis.

The results of this work point towards a vast intra-specific variability of emission rates for both, *H*. *halimifolium* and *C*. *ladanifer*, not only for kaurene but also for other isoprenoids, which is in good agreement with similar studies for other plant species^12,31,42,43^. Given that in the laboratory experiments all plants were kept under equal conditions, there might be a strong genetic plasticity among *H*. *halimifolium* individuals explaining the observed high variability. A high ecological^44,45^, phenological^33,46^ and genetic diversity^47^ has already been reported for the Cistaceae family. Therefore, as expected, *C*. *ladanifer* individuals differed also considerably in the magnitude of emissions, although under field conditions, other factors beyond genetic or phenotypic plasticity might have played a role. For instance the observed strong variability of kaurene emission rates by different individuals of the same species under field conditions could also be related to defence responses against pathogens and herbivores, as diterpenes have been found to act as phytoalexins, as constituents of oleoresins, or as activators of the systemic acquired resistance^22–24^. Furthermore, in the field, heat stress usually co-occurs with high radiation levels. Such excessive radiation levels constitute stress to plants under summer drought^41^; biosynthesis of kaurene could be involved in the prevention of photooxidation or light damage, by dissipating excessive energy from the electron transport chain into organic compounds. This is in line with the fact that *H*. *halimifolium* and *C*. *ladanifer* possess highly protective mechanisms against excessive irradiance and temperature^48^, as well as water stress^48^, similar to other Cistaceae species^44,48–50^. This study demonstrates that the kaurene released into the atmosphere by *H*. *halimifolium* and *C*. *ladanifer* seems to originate mainly from storage pools, and that plant internal kaurene contents together with prevailing temperature are most likely decisive drivers of emission rates. Nevertheless, the genetic or phenotypic plasticity of the Cistaceae family and their impact on modulating isoprenoid emissions together with the physiological and ecological function of kaurene in Cistaceae and other plant families deserve further attention.

*H*. *halimifolium* is a species able to successfully colonize dry and mesic dune habitats^51^, while *C*. *ladanifer* has a high spatial abundance^52^ and in the last decades, most probably due to environmental and land use changes, has rapidly expanded in drier regions^53^. Although we cannot state the ubiquitous character of the kaurene emissions in Mediterranean ecosystems, our results show that kaurene is emitted at least by two Cistaceae species, and therefore the atmospheric relevance of their diterpene emission should be investigated. Up to date, there is no information about possible atmospheric implications of diterpenes, as they were considered non-volatile and, thus, have not been studied in an atmospheric chemistry context. Despite the assumed low reaction rate constant of kaurene towards ozone and hydroxyl radicals of 1.2 × 10^−17^ cm^3^ molec^−1^ s^−1^ and 7.2 × 10^−13^ cm^3^ molec^−1^ s^−1^, respectively (EPI Suite, Environmental Protection Agency, USA), new studies should investigate the role of diterpenes in atmospheric chemistry and their potential to form secondary organic aerosols. Importantly, neglecting this type of compounds for atmospheric chemistry could also add to the so-called missing reactivity observed at some locations in the world^54–56^.

## Conclusion

Here, we demonstrated emissions of the diterpene kaurene under natural and laboratory conditions from two Cistaceae species and quantified emission rates by PTR-TOF-MS and TD-GC-MS. Both species revealed a strong individual variability in the magnitude of kaurene emissions, which were comparable to that of other emitted isoprenoids. Pyruvate position specific ^13^C-labelling indicated that emission of kaurene is most likely not derived from recently synthesized metabolites, whereas monoterpenes and sesquiterpenes revealed a partial *de novo* biosynthesis of labelled metabolites. Furthermore, concurrence of high leaf content and emissions of kaurene support the assumption that storage pools are the source of emission. This study demonstrates emissions of diterpenes by vegetation and indicates that this compound class may deserves further attention, similar as other, extensively studied, isoprenoids.

## Methodology

### Control experiments

#### Plant growth conditions

We measured a semi-malacophyll shrub from the Cistaceae family, *Halimium halimifolium L*., a typical Mediterranean dune shrub species, which is a main component of stable sand vegetation and is a generalist able to successfully colonize dry and mesic dune habitats^51^. All *H*. *halimifolium* individuals used for the diurnal courses of isoprenoid emissions (n = 7), labelling experiments of isoprenoid emissions (n = 10) and plant material extraction (n = 5) were grown from seeds in plastic pots (size: 15 × 15 × 11 cm) on a mixture of sand (1/3) and soil substrate (2/3) of medium structure in a greenhouse for 1.5 and 5 years. Plants were supplied with 100 ml of a modified Hoagland solution^46^ once a week^33^. Two months prior to the experiments, plants were transferred to a well-ventilated walk-in growth chamber, with fully controlled environmental conditions (ThermoTec, Weilburg, Germany). Light conditions were set to 500 µmol m^−2^ s^−1^ for 12 hours (9:00 to 21:00 h). Temperature and humidity were constant at 25 °C and 60%, respectively for all the experiments.

#### Methodology of measurements under controlled conditions

Measuring system of isoprenoid emissions and ecophysiological parameters: A newly designed measuring system for plant CO_2_ assimilation and BVOC emission measurements was used. This system provides 1 L of purified air, produced by a custom build zero air generator, to inert borosilicate glass cuvettes (volume 0.6 L). This air is free of oxidants and volatile organic compounds (VOC), and it is supplied at a known and constant humidity of 30% and CO_2_ mixing ratio of 400 ppm. Besides the inertness of the materials, all tubing lines (which were of same length for equal retention times) were made of perfluoroalkoxy (PFA) and were heated and isolated (50 °C) to avoid condensation and compound losses to tube walls. The sharp changes between plant and empty control cuvette in isoprenoid emissions (4 seconds) confirm minimum memory effect in the sampling lines. Determination of leaf isoprenoid emissions of *H*. *halimifolium* with this system took place in July and August 2016. At the cuvette outlet, air was drawn over to the PTR-TOF-MS, which was further connected to the differential infrared gas analyser (LI-7000 CO_2_/H_2_O Analyser; LI-COR, Lincoln, USA) for determination of leaf CO_2_ assimilation (A) and stomatal conductance (gs). Furthermore, air from the cuvette outlet was drawn (air sampling pump 210-1003MTX, SKC, Germany) over adsorbent tubes filled with 20 mg of Tenax TA 60/80 and 30 mg of Carbotrap B 20/40 for subsequent isoprenoid analysis by Thermodesorption-Gas chromatograph-Mass Spectrometer (TD-GC-MS). For this purpose, air samples were taken for 60 min at a flow of 200 cm^3^ min^−1^ (STP), leading to a collection of 12 L of cuvette air.

Diurnal courses of isoprenoid emissions by *H*. *halimifolium* were analysed under standard diurnal conditions (PPFD, 500 µmol m^−2^ s^−1^ during the light period; temperature, 25 °C during light and dark period) along with net CO_2_ assimilation and stomatal conductance in seven *H*. *halimifolium* individals. Diurnal courses were measured from 07:00 h to 23:00 h, where the light period was from 09:00 h to 21:00 h.

Plant isoprenoid extraction: Isoprenoids in leaf storage pools were determined by a modified method of Kallenbach *et al*.^57^. For this purpose, leaf samples were homogenized under liquid N_2_ and aliquots of 50 mg were added to 2000 µl methanol. Following 30 min of stirring at 30 °C and a subsequent centrifugation, five PDMS tube pieces (5 mm length) were put into 400 µl supernatant, which was diluted with 1600 µl H_2_O_demin_. Stirring occurred at 1,400 rpm for 60 min at 30 °C. After quantitative adsorption of isoprenoids onto PDMS-tubes, they were shortly dried with a lint free paper tissue, placed into glass tubes and analysed without further storage. Analysis occurred as described for air samples after TD-GC-MS.

Labelling scheme: Labelling studies were performed with position-specific ^13^C labelled pyruvate, a central metabolite of plant carbon metabolism with fast turn over times and a precursor for many BVOCs. ^13^C labelled pyruvate was fed via plant transpiration stream in order to evaluate if the emissions of isoprenoids were derived from *de novo* plant biosynthesis (i.e. emission of carbon that has been recently photosynthesized) or from other sources, such as storage pools. First, intact branches (put inside cuvette the day prior to measurement) of *H*. *halimifolium* were measured, then in the morning the branches were cut at the petiole. The branches were immediately recut under water and the stem was placed in deionized water. Two hours later the water solution was replaced by a ^13^C-labelled pyruvate solution (10 mM). Pyruvate was 99% labelled with ^13^C at the C_2_ carbon position (Sigma-Aldrich, Germany)^32,33^. Measurements were performed during three days in August 2016 and December 2017, in which two *H*. *halimifolium* individuals per day were measured sequentially. These experiments were also analysed under standard diurnal conditions (PPFD, 500 µmol m^−2^ s^−1^ during the light period; temperature, 25 °C during light and dark period) along with net CO_2_ assimilation and stomatal conductance measurements.

### Methodology of measurements in natural conditions

#### Field site description

Field measurements of kaurene fluxes were conducted in *Cistus ladanifer L*. shrubs growing in a cork oak (*Quercus suber* L.) woodland in Vila Viçosa (Alentejo, 38° 47′N, 7° 22′W, 430 m a.s.l.), Portugal, in June, July and August 2017 at different times of the day (from 08:00 h to 14:00 h). On each sampling day, three to five different twigs of the same individual were chosen for isoprenoid emission measurements. A total of four different *C*. *ladanifer* individuals were screened each month. Ecosystem variables such as air temperature, photosynthetically active radiation (PAR), solar radiation, air humidity, soil water content, VPD, precipitation, as well as eco-physiological parameters of the monitored plants were measured. Mean annual precipitation in the study site is 585.3 mm mostly distributed between October and May and mean temperatures reach 31.1 °C in July and 5.8 °C in January (mean annual temperatures of 15.9 °C) (Évora meteorological station, 1981–2010, http://www.ipma.pt, accessed in June 2017). More details of the site can be found elsewhere^53^.

#### Gas exchange measurements in the field

Selected twigs from the four chosen *C*. *ladanifer* individuals were enclosed in custom-made cuvettes (~460 ml) made of Nalophan foil and PFA, and connected via PFA tubing to air sampling pumps (210-1003MTX, SKC, Germany). These materials were chosen to minimize compound adsorption to tube surfaces due to their inertness. Care was taken that leaves were not damaged and did not touch the cuvette walls when enclosed. To acclimatize branches to the new conditions, cuvettes containing twigs with ca. 8 to 20 leaves were flushed for five minutes at a flow of 200 cm^3^ min^−1^ (STP); after that time CO_2_ assimilation rates and presumably also kaurene emission rates were stable. In addition, one empty cuvette was installed approx. 2 m above ground (at the same level as the plant cuvettes), and measurements from this cuvette were used for background correction for the plant cuvettes. The air supplied to the plant and empty control cuvettes was ambient air with no oxidant removal due to field measurement restrictions. We therefore denote that a possible underestimation of our fluxes in the field could be present due to ozonolysis and OH reactivity sink. For determination of kaurene emissions, air leaving the cuvettes were drawn over adsorbent tubes filled with polydimethylsiloxane- (PDMS) foam (Gerstel, Mülheim, Germany). Air samples were taken for 90 minutes in June and for 60 minutes in July and August at an air flow of 200 cm^3^ min^−1^ (STD).

During sampling, since the monitored branches were normally shaded by the upper canopy, a neutral-density shading mesh was placed above the cuvettes so shading conditions would prevail as constant as possible during measurements. In addition, adsorbent tubes were covered with aluminium foil during the sampling period. Immediately after sampling, twigs were cut and the leaves stored in a cooler for determination of leaf area and dry weight. Adsorbent tubes were stored in a fridge before taking them to the laboratory in Freiburg for TD-GC-MS analysis.

In addition, a portable photosynthesis system (LI-6400XT, LI-COR, Lincoln, USA) was used for A and gs measurements, made sequentially after isoprenoid sampling on leaves that were previously in the cuvettes. The recording frequency of the eco-physiological parameters was 1 second, and for better graphic representation, hourly averages were calculated for each measured plant.

### Analytical techniques

#### Proton transfer reaction - Time of flight – Mass spectrometer (PTR-TOF-MS)

In the laboratory studies, real-time measurements of isoprenoids were performed with a 4000ultra PTR-TOF-MS (Ionicon Analytic, Austria). The PTR-MS technique uses soft chemical ionization allowing for low fragmentation levels. This instrument uses an orthogonal acceleration electron time-of-flight mass spectrometer as detector, capable of measuring VOCs with high sensitivity (up to tens of cps/ppb), low detection limit (to ppt range), fast response time (less than 100 ms) and high mass resolution (5000 *m/Δm*)^58^. This newest 4000ultraPTR-TOF-MS model has some improvements such as a built-in internal mass calibration standard (diiodobenzene, *m/z* 330.848 and fragments on 203.943), which allows for the calibration of higher *m/z* scales. In addition, this version has an ion funnel at the end of the drift tube. The PTR-TOF-MS was operated at 2.7 mbar drift pressure, 600 V drift voltage, at an E/N of 120 townsend (Td), and drift tube heated to 80 °C. The recording frequency of the PTR-TOF-MS was 1 second, and for better graphic representation, hourly averages were calculated for each measured plant.

The PTR-TOF-MS data processing consisted in: i) correction for non-extending and extending dead times as well as the correction for Poisson statistics^59^ and iterative residual analysis and cumulative peak fitting^60^ using the DATA TOF ANALYZER software version 4.48; ii) normalization of the data to primary ions and water; (iii) background subtraction from the signal with the measurement of VOC-free air, due to impurities that originate from the system; and (iv) application of the calibration factors.

**Figure 6:**
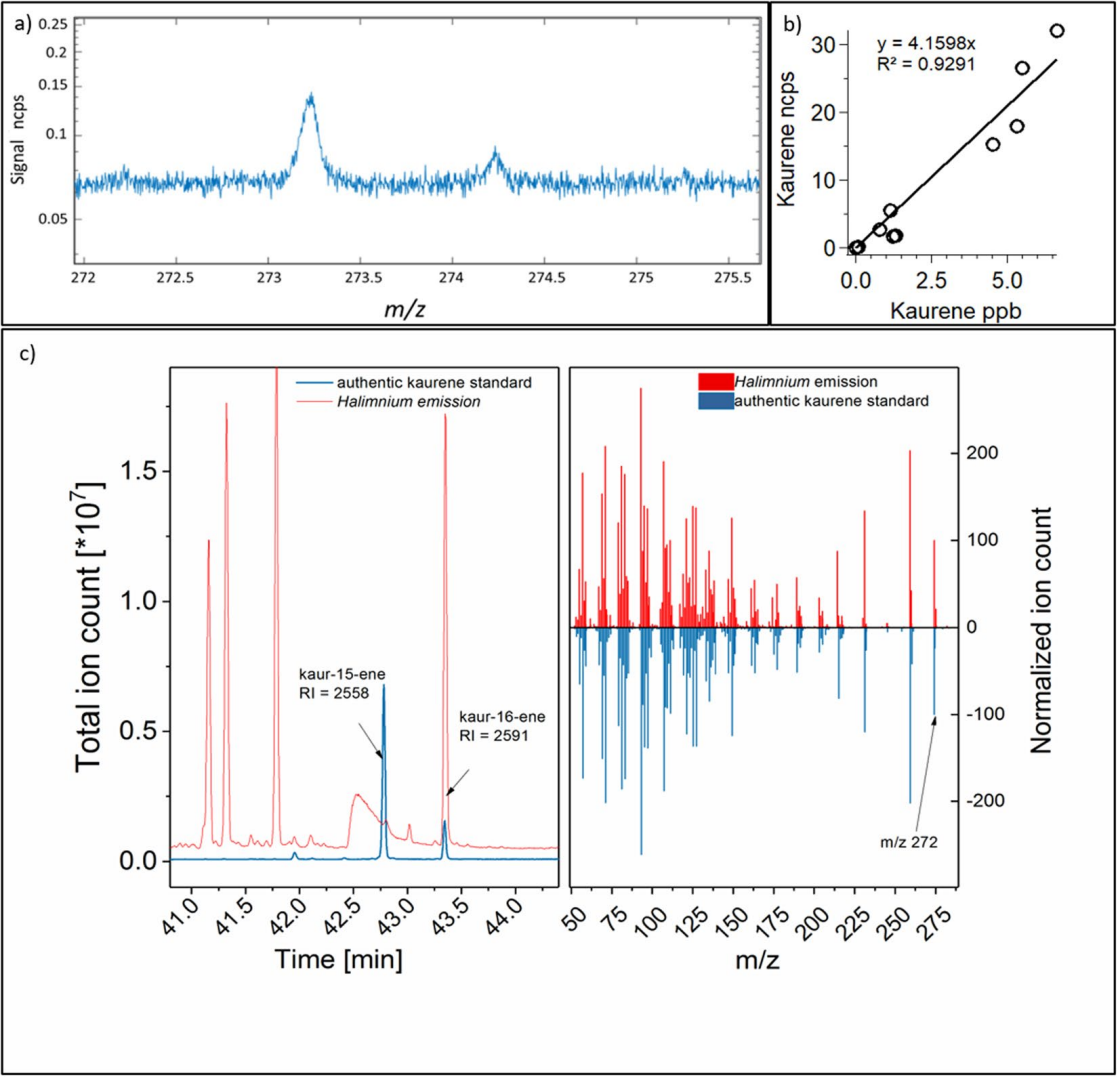
(**a**) Appearance of kaurene (^12^C compound and its first isotopologue) in the TOF spectra in normalized counts per second; (**b**) calibration of kaurene for the PTR-TOF-MS of the sum of the ^12^C compound and the first isotopologue in mixing ratio (ppb) against normalized counts per second; (**c**) the peak retention time for plant emissions (red) and standard (blue) kaurene for the TD-GC-MS (left panel), and comparison of TD-GC-MS fragmentation pattern for the plant emissions and standard of kaurene (right panel).

In the present study, we focused on *m/z* 69.07 (isotopologue *m/z* 70.07), *m/z* 137.13 (isotopologue *m/z* 138.13), *m/z* 205.20 (isotopologue *m/z* 206.20), which represent the compounds isoprene, monoterpenes and sesquiterpenes. We also detected substantial signals at *m/z* 273.20 and its isotopologue *m/z* 274.20 (Fig. 6a). By applying TD-GC-MS, we identified this peak at *m/z* 273.20 as kaurene (a.k.a. ent-Kaurene; kaur-16-ene, M_r_ 272.476 g mol^−1^), a tetracyclic diterpene, that exists in solid form and sublimes at room temperature (Fig. 6c)^19^. Once kaurene was identified from its mass spectrum, an authentic pure solid kaurene standard (OLChemIm Ltd., Olomouc. Czech Republic) was purchased and used to fully proof and quantify kaurene emission rates. The calibration for the PTR-TOF-MS including the calibration of the ^12^C molecule of kaurene and its isotopologue, is shown in Fig. 6b.

**Figure 7:**
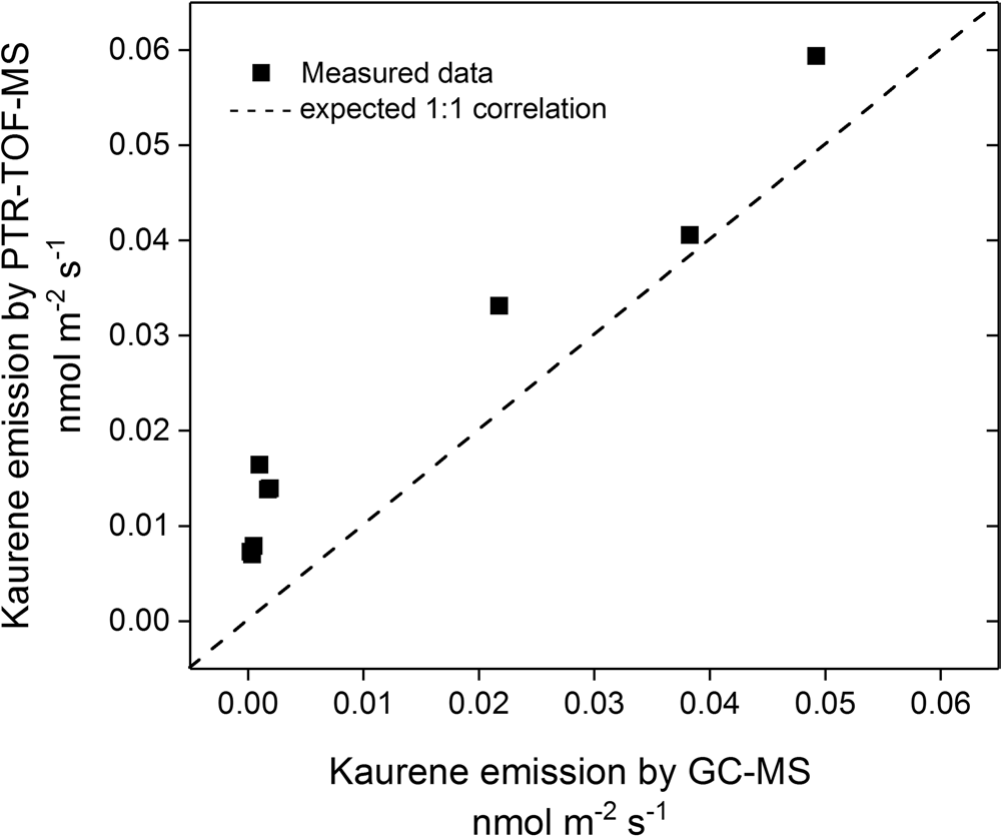
Relationship between PTR-TOF-MS and TD-GC-MS data. A 1:1 line is plotted for comparison.

The calibration factors of isoprene and α-pinene were obtained from humid calibrations from a gravimetrically prepared multicomponent gas standard (Ionicon Analytic, Austria). For β-caryophyllene (Sigma Aldrich, Germany) and kaurene liquid calibrations were performed. Both calibrations were done using the Liquid Calibration Unit (LCU, Ionicon Analytic, Austria), and since solutions were hexanal based, which is not soluble in water, humidity dependency could not be characterized for β-caryophyllene and kaurene. On the other hand, the comparability with the TD-GC-MS magnitudes gives us confidence on the accuracy of the calibration (Fig. 7). During PTR-TOF-MS measurements possible fragmentation may have occurred. However, since our measurements and calibrations were done at the same protonation conditions, the same fragmentation pattern can be expected for measurements and calibration, hence, providing a correct quantification under these protonation conditions. We tested for a possible fragmentation of myrcene on m/z 69.07 (fragmentation of 3.1%)^61^, however, the regression between m/z 137.13 and m/z 69.07 revealed a r^2^ of 0.17, evidencing no fractionation of myrcene on this mass. The total uncertainty of the PTR-TOF-MS determination was calculated according to the error propagation method taking into account the uncertainty of the calibration (including multicomponent gas standard and mass flow controller errors), the instrument error and the background error. The uncertainty of the measurements was determined to be 44% for isoprene, 24% for monoterpenes, 35% sesquiterpenes, and 46% for kaurene. In addition, detection limits were calculated as 3σ of the background averages. They were determined as 0.18 ppb for isoprene, 0.09 ppb for monoterpenes, 0.20 ppb for sesquiterpenes and 0.51 ppb for kaurene.

#### Thermodesorption- Gas chromatography – Mass spectrometer (TD-GC-MS)

All adsorbent tubes were analysed for isoprenoid content using a gas chromatograph (model 6890 A, Agilent Technologies Böblingen, Germany) equipped with a mass-selective detector (5975 C, Agilent Technologies Böblingen, Germany) and a thermodesorption-cold injection system (TDU-CIS) (Gerstel, Germany). Details are provided elsewhere^62^. The fragmentation spectra and available external standards of known concentrations were used for peak identification and quantification, respectively. Both, in field and laboratory studies, emission rates were calculated by subtracting isoprenoid mixing ratios of an empty control cuvette from plant cuvette concentrations and by accounting for the flow rate through the system and the leaf area in the cuvette.

#### Leaf area and dry weight calculation

The leaf areas of the branches in the cuvettes was determined using a scanner (CanoScan LiDE 110, Canon GmbH, Germany) and the GSA Imagine Analyser v4.09 (Software development and analytics GSA, GmbH, Germany). At the field site in Portugal, leaf area was determined with a customary scanner (EPSON EXPRESSION 1680) and analysed with the software WinSEEDLE (Regent Instruments Inc., Canada) in the laboratory. For comparability with other values reported in literature a conversion from nmol m^−2^ s^−1^ to µg g_dw_^−1^ h^−1^ using a dry weight-to-leaf area ratio of 63.28 obtained from the measurement of leaf areas and posterior dry weighting of such leaves was used.

### Calculations

#### Flux calculation

The equation used for the calculation of the fluxes was:

1 $$e=\frac{{u}_{i}}{s}\ast ({c}_{o}-{c}_{i})$$

where *u*_*i*_ is the molar flux in the cuvette inlet in mol s^−1^, *s* is the leaf area of measured branch in m^2^, c_o_ is the mixing ratio at the outlet of the cuvette and c_i_ is the mixing ratio at the cuvette inlet, both in mol mol^−1^.

#### Statistical analysis

Statistical significant differences between high and low emitter groups were assessed with a Kruskal-Wallies test as data failed the to meet the assumptions of normality and homeostasis by Shapiro-Wilk. In order to test if the isopreneoid emission rates and eco-physiological parameters for the control condition measurements were statistically significant regression analysis with a p-value of 0.05 were also performed. All statistical analyses were performed by the Sigma plot 2017 software (Systat, USA).

### Data availability statement

Data is available upon request to the main author.
